# Ba_2_BP_7_N_14_ – A Quaternary Alkaline Earth Nitridoborophosphate with a Mixed 3D Network Structure

**DOI:** 10.1002/chem.202404755

**Published:** 2025-02-21

**Authors:** Amalina T. Buda, Reinhard M. Pritzl, Monika M. Pointner, Jennifer Steinadler, Wolfgang Schnick

**Affiliations:** ^1^ Department of Chemistry University of Munich (LMU) Butenandtstraße 5–13 81377 Munich Germany

**Keywords:** boron, electron microscopy, high-pressure chemistry, mixed networks, quaternary nitridophosphates

## Abstract

Highly condensed alkaline earth nitridophosphates have attracted increasing scientific interest, due to their high thermal and chemical stability, as well as their promising luminescence behavior upon doping with Eu^2+^ for pc‐LED applications. In particular, the barely explored mixed tetrahedra‐based nitridophosphates offer a wide range of structural and compositional diversity, enabling new insights into structure‐property relationships. Herein, we report on the first quaternary alkaline earth nitridoborophosphate Ba_2_BP_7_N_14_, synthesized at 8 GPa and 1600 °C in a multianvil press, starting from Ba(N_3_)_2_, h‐BN and P_3_N_5_. Ba_2_BP_7_N_14_ crystallizes in the barylite‐1O polytype and features a highly condensed mixed (B,P)–N anionic 3D network (*κ*≈0.57) built up of PN_4_ and mixed occupied (P_0.75_B_0.25_)N_4_ tetrahedra. The structure was characterized by a multi‐step process involving single‐crystal and powder X‐ray diffraction (SCXRD, PXRD), elemental analysis, electron microscopy (STEM, EELS), and solid‐state ^31^P and ^11^B MAS NMR spectroscopy. The plausibility of the structural model was corroborated by low‐cost crystallographic calculations. The optical band gap and the thermal behavior of an undoped sample of Ba_2_BP_7_N_14_, were determined from diffuse reflectance spectroscopy and temperature‐dependent powder X‐ray diffraction, respectively. Irradiation of a Eu^2+^‐doped sample with near‐UV light results in a blue emission peaking at *λ*_em_=422 nm.

## Graphical abstract

The alkaline earth nitridoborophosphate Ba_2_BP_7_N_14_ was synthesized under high‐pressure high‐temperature conditions. The crystal structure reveals a 3D‐network of PN_4_ and mixed occupied (P_0.75_B_0.25_)N_4_ tetrahedra and was determined using a multi‐step characterization process including SCXRD, PXRD, elemental analysis, electron microscopy and NMR spectroscopy. Furthermore, materials properties were investigated. This approach provides first insights into mixed quaternary *AE*–B–P–N systems.

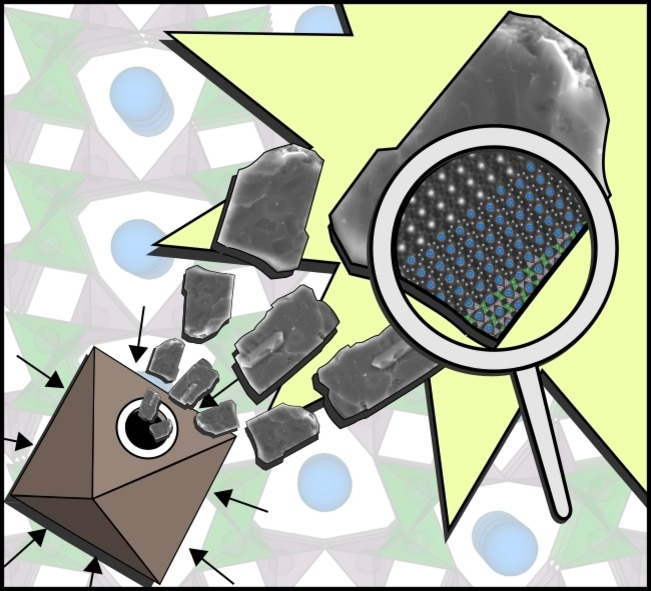

## Introduction

(Oxo)nitridophosphates are the subject of current research due to their structural diversity and the associated great potential for far‐reaching properties. Examples include the use of LiPON in solid‐state batteries in the context of energy conversion or the increasing industrial interest as phosphor materials in the field of solid‐state lighting (*SSL*).[^1^, ^2^, ^3^] The remarkable structural diversity is closely linked to the predominant structural motif of the PN_4_ tetrahedra. In nitridophosphates the latter can occur as isolated anions, small units or connected via common vertices or edges to chains, layers and 3D structures.[^4^, ^5^, ^6^, ^7^, ^8^, ^9^, ^10^, ^11^, ^12^, ^13^] The degree of condensation *κ* (defined as the ratio of network‐forming cation (*NFC*) to ligand (*LIG*)) is a characteristic measure for classifying, whether and to what extent the structures are cross‐linked. Especially highly condensed nitridophosphates (*κ*≥0.5) show growing scientific interest, due to their properties. In contrast to lowly condensed representatives like Li_4_PN_3_ (*κ*=1/3) or Sr_3_P_3_N_7_ (*κ*=3/7), highly condensed nitridophosphates such as LiPN_2_ (*κ*=1/2) or *M*P_8_N_14_ (*M*=Mg, Ca, Sr, Ba, Fe, Co, Ni; *κ*=4/7) are both thermally and chemically more stable.[^8^, ^9^, ^10^, ^14^, ^15^, ^16^] In particular, the mentioned alkaline earth metal (*AE*)P_8_N_14_ representatives have recently been increasingly investigated for possible applications. Upon doping with Eu^2+^, they exhibit promising ultra‐narrow‐band blue emission and have been discussed as potential candidates for pc‐LED applications.^[9]^ However, the degree of condensation *κ* of *AE* nitridophosphates and thus the structural diversity of highly condensed representatives is limited (*κ*_max_=3/5 in the binary parent compound P_3_N_5_).^[17]^ To further expand the compositional and structural versatility, the range of *κ* can be extended or the cation ratio (*CR*=counter cation (*CC*)/*NFC*) can be varied, as discussed in literature.[^18^, ^19^] Both cases can be achieved by incorporating additional *NFC*s into the anionic tetrahedral network. This strategy has been successfully demonstrated for the classes of *AE* (oxo)nitridosilicatephosphates and *AE* nitridomagnesophosphates, which consist of mixed tetrahedral (Si,P)−(N,(O)) and (Mg,P)−N networks, respectively.[^19^, ^20^, ^21^, ^22^] The hitherto highest degree of condensation in quaternary mixed *AE* nitridophosphates was observed for SrSi_2_PN_5_ and Ba_3−*x*_Sr_*x*_[Mg_2_P_10_N_20_] (*x*=0–3), with *κ*=0.6.[^20^, ^22^] In the latter, the formation of an unprecedented structure type was observed. Furthermore, significantly different structures are evident in Sr_2_SiP_2_N_6_ and Sr_5_Si_2_P_6_N_16_, compared to the ternary compound SrP_2_N_4_, which crystallizes in the megacalsilite‐type structure.[^11^, ^19^] These differences arise due to their distinct *CR* values (2/3, 5/8, and 1/2, respectively) despite sharing the same *κ* value of 1/2. This structural diversity has also provided a deeper understanding of the structure‐property relationship of *AE* nitridophosphate related compounds, especially regarding their luminescence properties. However, the investigation of quaternary *AE* nitridophosphates with other mixed anionic tetrahedral networks remains largely unexplored.

In this context, the *AE*–B−P−N system presents a promising research opportunity. Inspired by the superhard cubic boron nitride (c‐BN), where the covalent B−N bonds within BN_4_ tetrahedra contribute to exceptional thermal and chemical stability, the integration of BN_4_ units into the anionic PN_4_ tetrahedra network could potentially yield comparable advantages.[^23^, ^24^] The feasibility of combining P−N with B−N system has been demonstrated in the past by the compounds Li_47_B_3_P_14_N_42_ and *α*‐/*β*‐BP_3_N_6_.[^25^, ^26^, ^27^] The lithium nitridoborophosphate features three different anion types: two pure nitridophosphate‐type anions [P_4_N_10_]^10−^ and [P_3_N_9_]^14−^, and one mixed [P_3_B_3_N_13_]^15−^ anion, which consists of three trigonal planar BN_3_ units and three PN_4_ units. Similar to the anionic structural motifs in nitridoborates, the BN_3_ units can be derived from hexagonal boron nitride (h‐BN) where the boron atoms are *sp* ^2^ hybridized.[^25^, ^28^] In contrast, both modifications of BP_3_N_6_ exhibit BN_4_ tetrahedra with *sp* ^3^ hybridized B atoms, which can be derived from c‐BN and are rarely observed in nitride compounds. In the double nitride *α*‐BP_3_N_6_, these form a highly condensed mixed (B,P)−N tetrahedral network, resulting in great thermal and mechanical stability.[^26^, ^27^] Beyond Li_47_B_3_P_14_N_42_ and the mixed non‐metal nitride BP_3_N_6_, no additional representatives within the B−P−N system have been identified to date.

This limitation can be attributed to the challenging synthesis conditions required to combine both, the P−N and B−N substance classes. Typically, nitridoborates and nitridophosphates are prepared starting from their binary parent compounds (h‐BN and P_3_N_5_). Therefore, high temperatures are required to ensure bond cleavage and reformation. However, P_3_N_5_ decomposes at temperatures above 850 °C, whereas significantly higher temperatures are required to activate h‐BN.[^17^, ^24^] In accordance with Le Chatelier′s principle, the application of pressure can prevent thermal dissociation of P_3_N_5_.^[4]^ Elevated pressure is also required to increase the coordination number (*CN*) of boron and promote the formation of BN_4_ tetrahedra. This is evident in the phase transition from h‐BN (*CN*=3) to c‐BN (*CN*=4) under high‐pressure high‐temperature (HP/HT) conditions.[^23^, ^29^]

Herein we present the successful synthesis and characterization of the first alkaline earth nitridoborophosphate Ba_2_BP_7_N_14_, containing a highly condensed mixed (B,P)−N tetrahedral network. A multistep characterization process (X‐ray diffraction, elemental analysis, low‐cost crystallographic calculations, as well as advanced STEM‐HAADF and solid‐state NMR analyses) enables the successful investigation of the crystal structure. In addition, material properties of this new substance class were investigated including thermal stability, optical band gap, and luminescence behavior upon doping with Eu^2+^.

## Results and Discussion

### Synthesis

The title compound Ba_2_BP_7_N_14_ was serendipitously discovered when the crucible material (h‐BN) unintentionally reacted with the starting material mixture (Ba(N_3_)_2_ and P_3_N_5_) during a HP/HT nitridophosphate synthesis. Appropriate crystals suitable for single‐crystal X‐ray diffraction (SCXRD) were successfully isolated. Previous synthesis experiments using a platinum capsule have demonstrated that the phase identified by SCXRD only forms in the presence of h‐BN (Supporting Information Figures S1, S2). Furthermore, it was observed that the use of h‐BN powder positively influences the increase in phase content, as the sintered h‐BN (crucible material) demonstrates a higher degree of inertness towards the employed reactants. Based on these findings, an optimized synthesis for the title compound was developed.

Ba_2_BP_7_N_14_ was synthesized under HP/HT conditions at 8 GPa and 1600 °C, using a Walker‐type multianvil press. Stoichiometric amounts of the metal azide Ba(N_3_)_2_ and the respective binary nitrides (P_3_N_5_ and h‐BN) were used as starting materials according to equation 1:
(1)

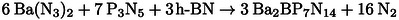




**Figure 1:**
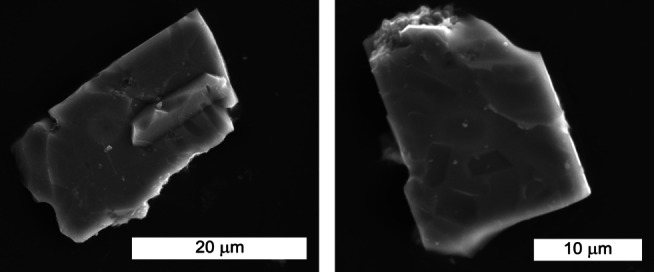
SEM images of Ba_2_BP_7_N_14_. Block‐like single‐crystals with a maximum diameter of 30 μm were observed.

For luminescence investigations, EuCl_2_ was added as dopant (1 mol% with respect to the Ba^2+^ content). Further details on the HP/HT synthesis are provided in the Experimental Section and the Supporting Informations (Table S1). The title compound was obtained as air‐ and moisture‐stable, colorless powder. Block‐like single‐crystals with an edge length of up to 30 μm were observed by scanning electron microscopy (SEM, Figure 1).

### Crystal Structure Determination

The crystal structure of Ba_2_BP_7_N_14_ was solved and refined from SCXRD data in the orthorhombic space group *Pmn*2_1_ (no. 31, *a*=11.9833(3), *b*=4.94300(10), *c*=4.74010(10) Å, *Z*=1).^[30]^ During the initial refinement process, two distinct phosphorus sites were identified: a fully occupied (P1) and a partially occupied (P2) site. Under the premise of charge neutrality, the reactants used and potential minor elements O and H (which often cannot be completely excluded in high‐pressure synthesis), the following three possible sum formulas result: “Ba_2_P_7_N_11_O_3_”, “Ba_2_P_7_N_11_(NH)_3_” and “Ba_2_BP_7_N_14_”. Consequently, the P2 site in “Ba_2_P_7_N_11_O_3_” and “Ba_2_P_7_N_11_(NH)_3_” would remain partially occupied, whereas in “Ba_2_BP_7_N_14_” this site would be mixed occupied with boron (P2/B2).

Energy dispersive X‐ray (EDX), elemental combustion (CHNS), and solid‐state magic angle spinning nuclear magnetic resonance (MAS NMR) analyses were carried out to determine the correct elemental composition. Based on EDX measurements on individual crystals, the atomic ratio of Ba:B:P:N:O≈2:2:7:13:1 was obtained (Table S2). It should be noted that the amount of lighter atoms (B/N/O) is usually over‐ or underestimated in the presence of heavier atoms. However, the atomic ratio of Ba and P agrees well with the ratio obtained from the single‐crystal model. CHNS analysis revealed a nitrogen content of 27.4 wt%, whereby no hydrogen was detected (Table S3). Since both methods give a higher nitrogen value than expected for the possible oxygen‐rich “Ba_2_P_7_N_11_O_3_” (N_theo._: 22.2 wt%), this composition was ruled out. Low oxygen content measured by EDX may be due to minor surface hydrolysis. Furthermore, since no hydrogen was observed in the CHNS analysis and no signals were detected in the ^31^P{^1^H} cross polarization MAS NMR experiment, the possible sum formula Ba_2_P_7_N_11_(NH)_3_ was finally excluded (see Supporting Information). Due to the necessity of h‐BN for the formation of the observed compound (see synthesis section) and the results mentioned above, the sum formula Ba_2_BP_7_N_14_ can be assumed. As a result, in the SCXRD structure refinement process, a substitutional disorder of the P2 site by B was refined. The free, restrained refinement leads to a P:B ratio of 0.75(P2):0.25(B2). To exclude possible ordering variants the structural model was additionally solved and refined in space group *P*1. Since P/B disorder was also observed at all four positions (Wyck. position 4*b* splits into 4 x 1*a*) within the standard deviations, a possible ordering variant was ruled out. Detailed information on the crystallographic data, Wyckoff positions, atomic coordinates, anisotropic displacement parameters, interatomic distances and angles are given in Tables 1 and S4–S7. Due to the low scattering contrast of boron in X‐ray diffraction, additional analyses, including electron energy loss spectroscopy (EELS), scanning transmission electron microscopy with high‐angle annular dark‐field (STEM‐HAADF), and ^11^B and ^31^P MAS NMR experiments were performed to further investigate and confirm the unusual mixed P/B site. Detailed information can be found in the corresponding chapters.

**Table 1:** Crystallographic data of single‐crystal refinement of Ba_2_BP_7_N_14_. Standard deviations are given in parentheses.

Formula	Ba_2_BP_7_N_14_
Crystal system	orthorhombic
Space group	*Pmn*2_1_ (no. 31)
Lattice parameters / Å	*a*=11.9833(3) *b*=4.94300(10) *c*=4.74010(10)
Cell volume / Å^3^	280.772(11)
Formula units per unit cell	1
Density / g⋅cm^−3^	4.131
*μ* / mm^−1^	7.996
*T*_min_ */T*_max_	0.8935/1.0000
Radiation	Mo‐K*α* (*λ*=0.71073 Å)
Temperature / K	297(2)
*F*(000)	320
*θ* range / °	4.123 < *θ* < 36.300
Total no. of reflections	11685
Independent reflections (> 2σ)	1400 (1385)
Refined parameters	59
Flack parameter	0.044(12)
BASF	0.0236^[a]^
*R*_int_; *R*_σ_	0.0239; 0.0155
*R*1 (all data); *R*1 (*F* ^2^ > 2σ(*F* ^2^))	0.0179; 0.0176
*wR*2 (all data); *wR*2 (*F* ^2^ > 2σ(*F* ^2^))	0.0381; 0.0380
Goodness of fit	1.141
Δ*ρ*_max_; Δ*ρ*_min_ / e ⋅ Å^−3^	2.00; −1.78

[a] For refinements, an inversion twin was considered.

**Figure 2:**
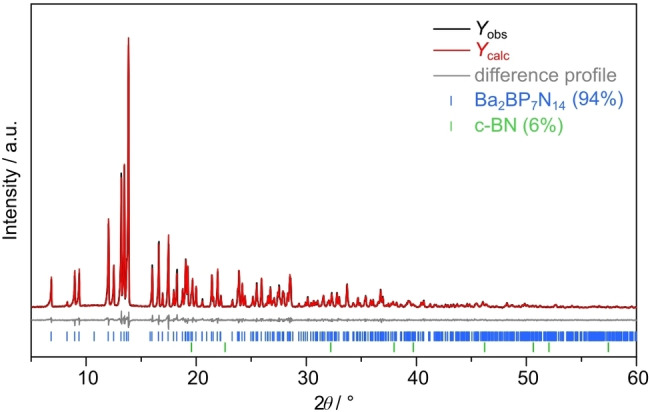
Rietveld refinement based on PXRD data of Ba_2_BP_7_N_14_; observed (black line) and calculated (red line) powder X‐ray diffraction patterns, difference profile (gray line), positions of Bragg reflections of Ba_2_BP_7_N_14_ (94 wt%, vertical blue bars) and c‐BN (6 wt%, vertical green bars).

Rietveld refinement based on powder X‐ray diffraction (PXRD) data confirmed the structure model obtained from SCXRD data and was used to analyze the phase content of the title compound in bulk material (Figure 2, Tables S8, S9). Besides Ba_2_BP_7_N_14_, only c‐BN (6 wt%) was identified as a minor side phase.

### Structure Description

**Figure 3:**
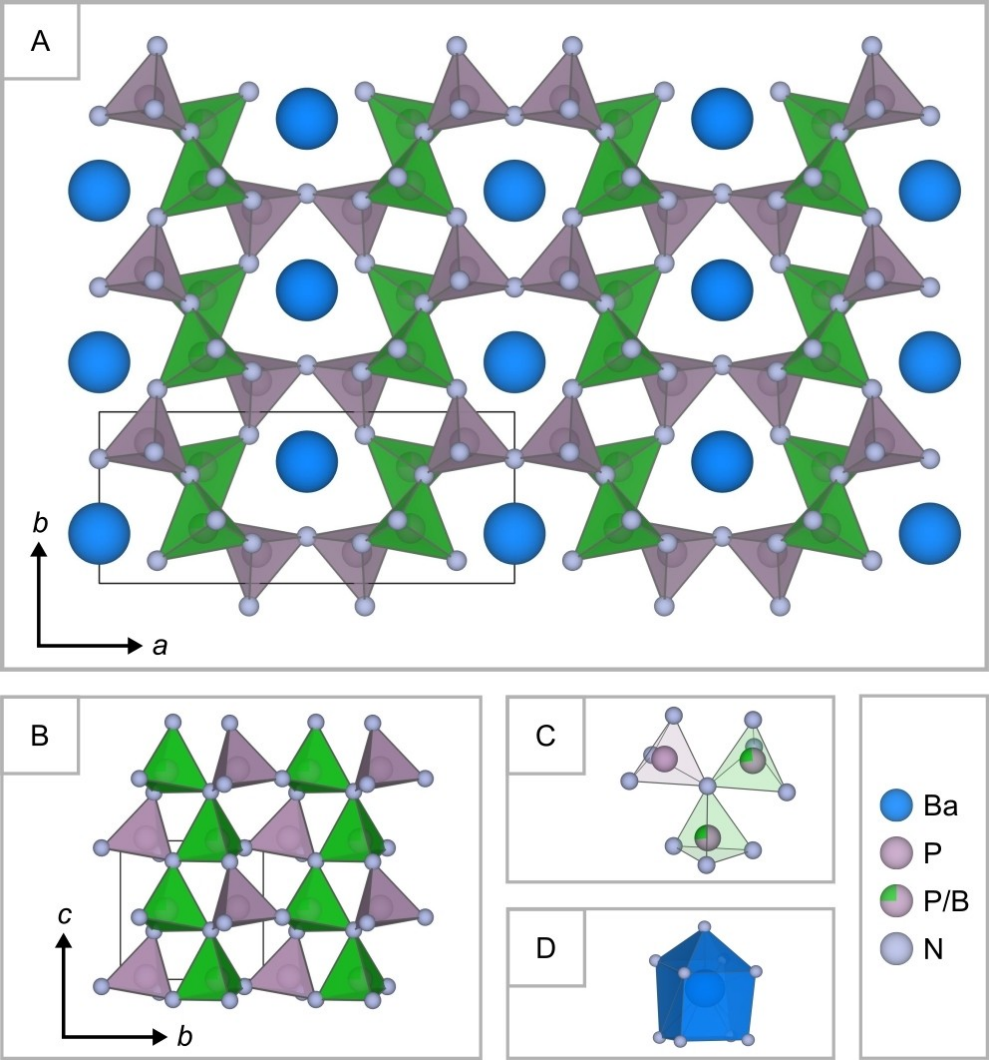
Crystal structure of Ba_2_BP_7_N_14_. A: Three‐dimensional network consisting of all‐side vertex‐sharing PN_4_ (purple) and (P_0.75_B_0.25_)N_4_ tetrahedra (green), forming *vierer‐* and *sechser‐*rings along [001]. Ba^2+^ ions (blue) are located in the *sechser*‐ring channels. B: All tetrahedral vertices are aligned parallel to *c*. C: Propeller‐like subunits composed of one PN_4_ and two (P_0.75_B_0.25_)N_4_ tetrahedra, which are connected by a threefold bridging N^[3]^ atom. D: BaN_9_ polyhedra (blue) forming a distorted variation of an elongated square pyramid.

Ba_2_BP_7_N_14_ can be classified as a highly condensed *AE* nitridoborophosphate with the same *κ*≈0.57, as the ternary *AE* and transition metal nitrodophosphates *M*P_8_N_14_ (*M*=Mg, Ca, Sr, Ba, Fe, Co, Ni).[^8^, ^9^, ^10^] In contrast to these layered compounds, Ba_2_BP_7_N_14_ crystallizes in the barylite‐1O polytype (MOD_1_, BaBe_2_Si_2_O_7_) and forms a 3D network structure.^[31]^ This is composed of all‐side vertex‐sharing PN_4_ and mixed occupied (P_0.75_B_0.25_)N_4_ tetrahedra (Figure 3A) and can be described by the point symbol {3^2^.4^3^.5.6^4^}{3^4^.4^5^.5^4^.6^2^} using TOPOS.^[32]^ Ba_2_BP_7_N_14_ is isotypic to the compounds *AE*SiP_3_N_7_ (*AE*=Sr, Ba; *Pmn*2_1_), which also exhibit one fully occupied P site and one mixed occupied (P_0.5_Si_0.5_) site.^[33]^ Thus, both networks contain atoms with different oxidation states at the same crystallographic position ((B^+III^/ P^+V^); (Si^+IV^/ P^+V^); Wyck. position 4*b*). Disordered network structures are not uncommon in nitrides, as observed in compounds such as Sr[Mg_2_Ga_2_N_4_], Sr[Mg_3_GeN_4_], and Sr_*x*_Ca_1−*x*_[AlSiN_3_].[^34^, ^35^] These feature mixed occupied (Mg^+II^/ Ga^+III^), (Mg^+II^/ Ge^+IV^), and (Al^+III^/ Si^+IV^) tetrahedral positions, respectively.

As described above, Ba_2_BP_7_N_14_ crystallizes in the MOD_1_ polytype. In contrast to the MDO_2_ polytype, all tetrahedral vertices in the title compound are oriented in the same direction (Figure 3B).^[31]^ The tetrahedra are connected by N^[2]^ and N^[3]^ atoms, with the latter forming propeller‐like subunits (Figure 3C). The P−N and (P/B)−N distances for N^[2]^ are in the range of 1.593(3)−1.603(2) Å and 1.632(3)−1.644(3) Å, respectively, which are slightly shorter than those for N^[3]^ (1.684(3)) Å, and 1.729(3)−1.739(4) Å, respectively). These values are consistent with those observed for related compounds.[^9^, ^26^, ^33^, ^36^] The Ba^2+^ ions are located within the *sechser*‐ring channels and forming BaN_9_ polyhedra, which can be descripted as a distorted variation of an elongated square pyramid (Figure 3A & D).[^37^, ^38^] The Ba−N distances (2.815(3)−3.200(3) Å) correlated well with the *AE*−N distances of the related compounds *AE*SiP_3_N_7_ (*AE*=Sr, Ba).^[33]^

### Low‐Cost Crystallographic Calculations

The electrostatic plausibility of the structural model of Ba_2_BP_7_N_14_ was examined through charge distribution (CHARDI) and Madelung part of the lattice energy (MAPLE) calculations.[^39^, ^40^] CHARDI indicates a mean total charge of +1.94 (Ba), +4.53 (P_0.75_B_0.25_), +5.00 (P) and −2.80 to −3.14 (N) and effective coordination numbers of 3.87 (P/B) and 3.92 (P) for the *NFC*s, which agree well with the theoretical values. The overall MAPLE value of Ba_2_BP_7_N_14_ (199896 kJ· mol^−1^) is also in good agreement with the sum of the MAPLE values of the formally constituting ternary phases BaP_2_N_4_ and BP_3_N_6_ (202494 kJ· mol^−1^) with 1.3% deviation. The results of the low‐cost crystallographic calculations thus support the refined structure model from SCXRD. Detailed information about CHARDI and MAPLE is provided in the Supporting Information (Tables S10, S11).

### Electron Energy Loss Spectroscopy (EELS) and Scanning Transmission Electron Microscopy (STEM)

As demonstrated by the characterization of *AE* nitridosilicatephosphates, such as BaSiP_3_N_7_ and Sr_5_Si_2_P_6_N_16_, STEM is a valuable method for elucidating disordered structures.[^19^, ^33^] Therefore, STEM and EELS analyses were performed on suitable crystallites of Ba_2_BP_7_N_14_ to gain further insight into the occupancy of the *NFC*s within the crystal structure. The EELS spectra (Figures S3, S4) show the P‐L_2,3_ edge at ~137 eV energy loss, which is consistent with the determined value of the investigated reference material BaP_2_N_4_. Moreover, the B‐K edge was identified at an energy loss of ~194 eV in the spectra of Ba_2_BP_7_N_14_. This value is in a good agreement with the B‐K edge of c‐BN as determined in literature.^[41]^ The results of the EELS analysis confirm the presence of both elements P and B in the crystal structure of Ba_2_BP_7_N_14_, as assumed in SCXRD analysis.

**Figure 4:**
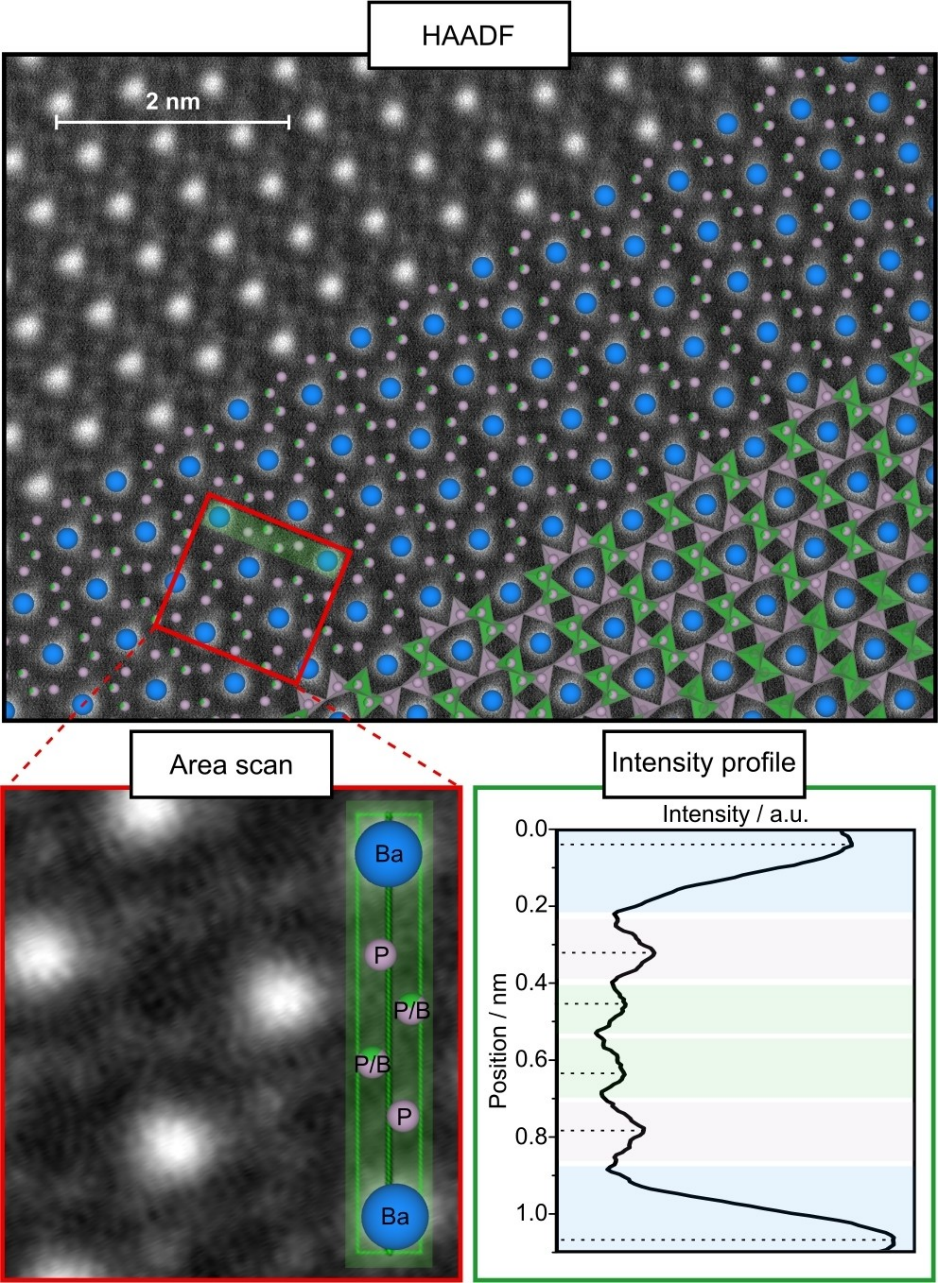
STEM‐HAADF image with structure overlay of Ba_2_BP_7_N_14_ along [001] (top). Area scan (bottom left) over the atomic positions Ba–P–P/B–P/B–P–Ba and resulting intensity profile (bottom right) with Ba (blue), P (purple) and P/B (green). The intensity profile shows a significant difference between the fully occupied P and the mixed occupied P/B site.

STEM‐HAADF images were recorded along the zone axes [001] with a *Z*‐contrast according to *Z* ^2^ (Figure 4 (top), S5).^[42]^ In this orientation, the atomic columns are separated, allowing for the distinction and individual evaluation of the mixed P2/B2 and the fully occupied P1 site. Consequently, several regions of the atomic occupancy of Ba–P–P/B–P/B–P–Ba were investigated using two‐dimensional intensity profiles (Figure 4 (bottom)). In contrast to the P1 site, significantly lower intensity maxima were observed for the disordered P2/B2 site. In order to quantify the occupation ratio of P/B site, the percentage ratio of the normalized intensity maxima of this site to P1 (100%) was determined. The mixed occupancy indicates an intensity ratio of 71%, corresponding to an atomic occupancy of P:B≈0.7:0.3. Detailed results and calculations are given in the SI (Tables S12, S13 and Equations S1–S3). Considering the statistical distribution of P and B on this site, the determined occupancy ratio, agrees well with that obtained by the refined structure model.

### Solid‐State Nuclear Magnetic Resonance (NMR) Spectroscopy

**Figure 5:**
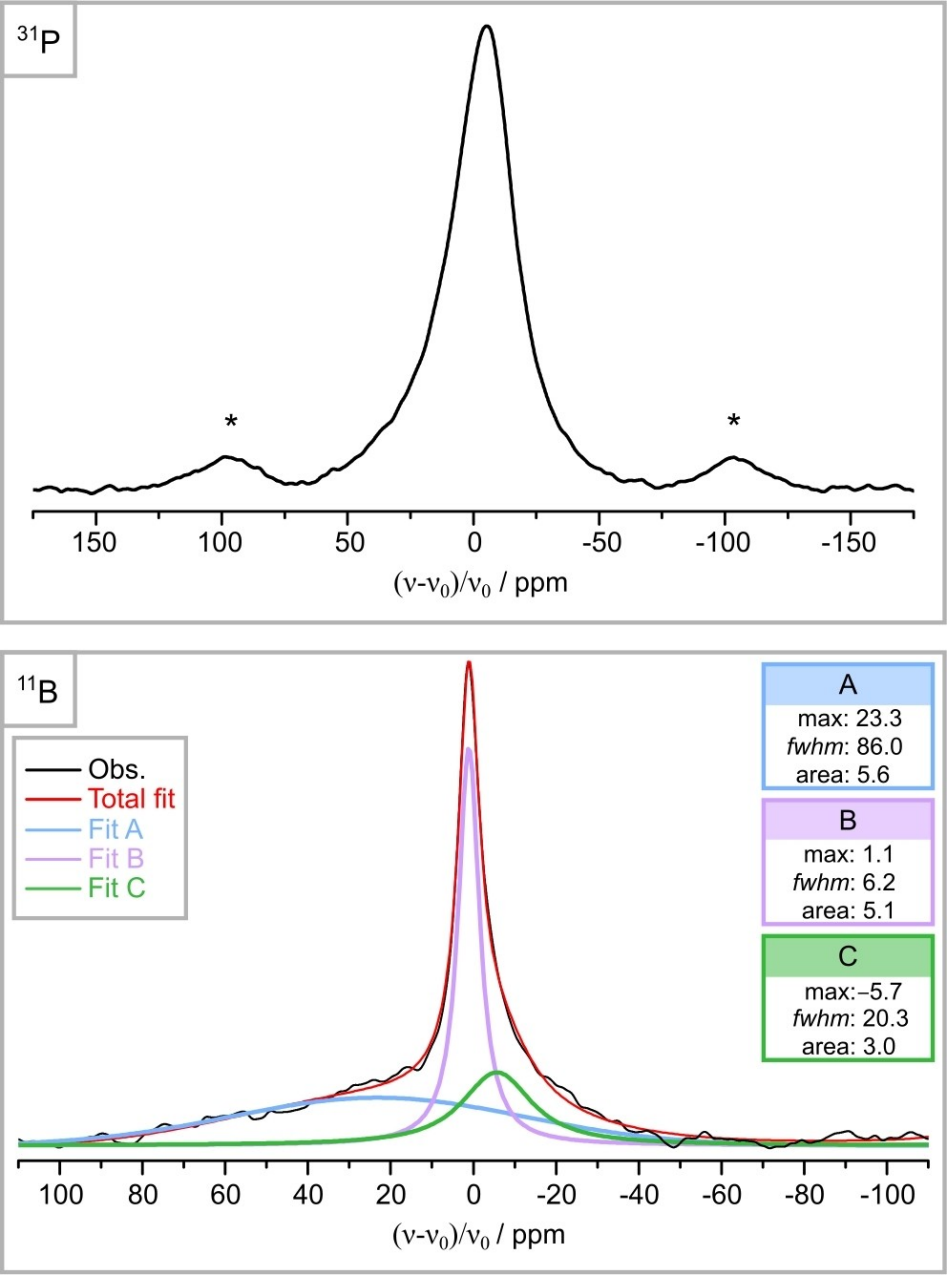
^31^P MAS NMR (top) and ^11^B spin‐echo MAS NMR (deconvolution, bottom) spectra of Ba_2_BP_7_N_14_. The ^31^P MAS NMR spectrum shows a broad signal at −5.3 ppm, which can be attributed to an overlap of the two expected ^31^P signals. The signals indicated by an asterisk were identified as rotational sidebands. The deconvolution fit (red) of the observed ^11^B NMR spectrum (black) contains three signals at 23.3 (A, blue), 1.1 (B, purple) and −5.7 ppm (C, green). They can be assigned to h‐BN (amorphous), c‐BN and Ba_2_BP_7_N_14_, respectively.

Solid‐state ^31^P MAS NMR and ^11^B spin‐echo MAS NMR experiments were performed, to confirm the obtained structure model from X‐ray diffraction data and to investigate the bulk material. The ^31^P MAS NMR spectrum in Figure 5 (top) shows one broad signal with *fwhm*=28.7 ppm at *δ*=−5.3 ppm. Due to the ordered P1 and mixed occupied P2/B2 site (both Wyck. position 4*b*), two NMR signals are expected. The observed broad signal can be attributed to a broadening effect of the statistically disordered P2/B2 site, resulting in an overlap of the two expected ^31^P signals. However, a deconvolution of the distinct signals was not possible. A broadening of the ^31^P signals from disordered *NFC* positions has already been discussed for the compounds Sr_3_SiP_3_O_2_N_7_, Sr_5_Si_2_P_4_ON_12_ and *AE*SiP_3_N_7_ (*AE*=Sr, Ba), supporting the chosen structure model.[^21^, ^33^]

In order to obtain ^11^B signals from the sample and to eliminate background signals from the NMR hardware, a ^11^B spin‐echo MAS NMR experiment was performed.^[43]^ For the deconvolution of the measured ^11^B spectrum (Figures S7, S8), at least three signals were required to properly reproduce the experimental data in the region between 100 and −100 ppm (Figure 5 (bottom)). The broad signal at 23.3 ppm (A, blue) and the major narrow signal at 1.1 ppm (B, purple) can be attributed to side phases h‐BN (amorphous) and c‐BN, respectively.[^44^, ^45^] The signal at −5.7 ppm (C, green) can be assigned to Ba_2_BP_7_N_14_. The chemical shift at −5.7 ppm is consistent with the typical range for tetrahedrally coordinated B atoms.[^26^, ^45^, ^46^] Furthermore, the broadening of this signal is consistent with the statistically mixed occupation of the P_0.75_B_0.25_ site and supports the SCXRD structure model.

In order to ascertain the consistency of the NMR data with that obtained via powder X‐ray diffraction, the area ratio of the ^11^B NMR signals and the molar ratio of the B atoms from Rietveld refinement of c‐BN and Ba_2_BP_7_N_14_ were compared (Table S14). In both cases, the ratio of c‐BN to Ba_2_BP_7_N_14_ was approximately 2:1, which implies that both analytical methods provide comparable results for the bulk material. However, since this determination does not consider the differences in sample volumes used by the two methods, it can only be regarded as a qualitative result.

### Temperature‐Dependent Powder X‐Ray Diffraction

**Figure 6:**
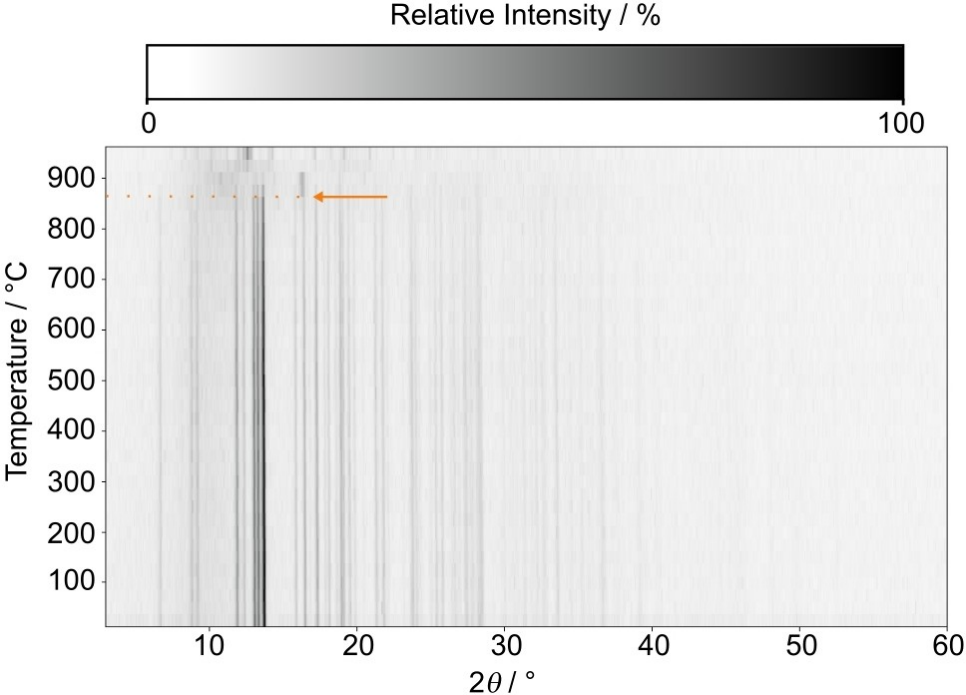
Temperature‐dependent powder X‐ray diffraction patterns (Mo‐K*α*_1_ radiation, *λ*=0.7093 Å) of Ba_2_BP_7_N_14_, showing a moderate thermal expansion with increasing temperature, and a starting decomposition at 875 °C.

In order to investigate the thermal behavior of Ba_2_BP_7_N_14_, temperature‐dependent powder X‐ray diffraction patterns were recorded between 25–950 °C in air. Figure 6 shows no evidence of phase transition or decomposition up to 850 °C. A moderate thermal expansion (average 7.5(5) ppm/K) was determined within the range of 25–850 °C (Figure S9). Above 850 °C a decomposition process was observed, resulting in the complete absence of Ba_2_BP_7_N_14_ at temperatures ≥ 925 °C.

### Optical Properties – UV/Vis Reflectance and Luminescence Spectroscopy

**Figure 7:**
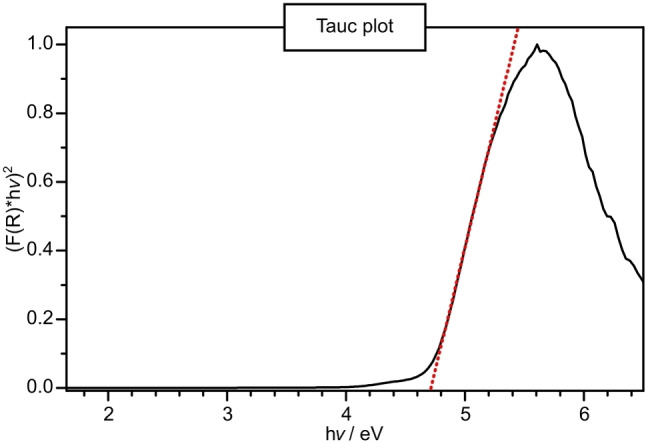
Tauc plot (black line) for non doped Ba_2_BP_7_N_14_ with tangent (red dotted line) at the inflection point. A optical band gap of ≈4.7 eV can be estimated (intersection of x‐axis and tangent).

The optical properties were examined by diffuse reflectance (undoped sample) and luminescence (with ~ 1 mol% Eu^2+^ doped sample: Ba_2_BP_7_N_14_:Eu^2+^) measurements. The UV/Vis spectrum exhibits an absorption band around 300 nm (Figure S10). The reflectance spectrum was transformed into a pseudo‐absorption spectrum through the application of the Kubelka‐Munk function.^[47]^ The experimental band gap was determined by plotting *hν* versus (F(R) ⋅ *hν*)^1/*n*^, assuming a direct band gap (*n*=1/2) and drawing a tangent at the inflection point of the Tauc plot (Figure 7).^[48]^ The optical band gap was thus estimated to ≈4.7 eV, which is in line with the colorless bulk material.

**Figure 8:**
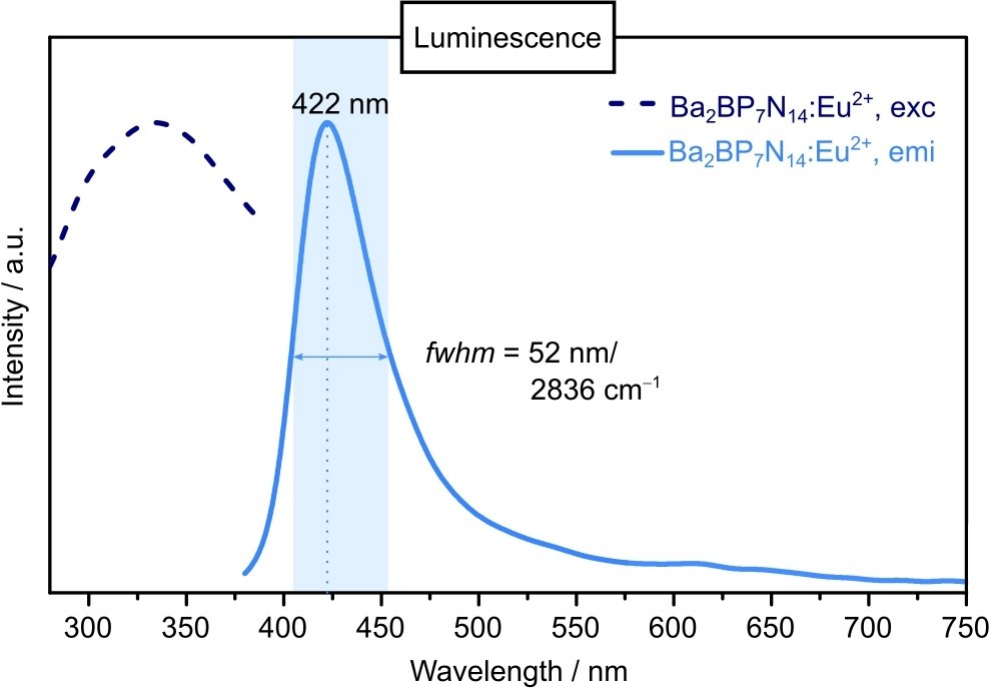
Luminescence spectra of Ba_2_BP_7_N_14_:Eu^2+^ bulk material. Normalized excitation (*λ*_exc_=334 nm, dark blue) and emission spectra (blue) with an emission maximum at 422 nm (*fwhm*=52 nm/2836 cm^−1^).

Due to the promising wide band gap of ≥ 4 eV required for pc‐LED applications, the luminescence properties of bulk Ba_2_BP_7_N_14_:Eu^2+^ were studied. Therefore, excitation and emission spectra were recorded. Excitation with near‐UV light (*λ*_exc_=334 nm) exhibits a blue emission at *λ*_em_=422 nm (*fwhm*=52 nm/2836 cm^−1^, Figure 8). Due to the relatively small ionic radii of the *NFC*s, the Ba^2+^ site is favored by Eu^2+^ doping (*r*(B^3+^)=0.11 ppm, *r*(P^5+^)=0.17 ppm, *r*(Ba^2+^)=1.47 ppm, *r*(Eu^2+^)=1.3 ppm).^[49]^ The observed emission band can thus be attributed to Eu^2+^ at the single Ba^2+^ site. Similar emission maxima and half widths were determined for the related compounds SrSiP_3_N_7_:Eu^2+^ (*λ*_em_=430 nm, *fwhm*=45 nm/2404 cm^−1^; *Pmn*2_1_) and BaSiP_3_N_7_:Eu^2+^ (*λ*_em_=424 nm, *fwhm*=53 nm/2731 cm^−1^; *Pnma*).^[33]^ This observation can be explained by similar bond distances (*d*_Ø_(Sr−N)=2.9686 Å, *d*_Ø_(Ba−N)=3.0026 Å) and environment of the *AE* ^2+^ cations of the latter compounds to Ba_2_BP_7_N_14_ (*d*_Ø_(Ba−N)=3.0024 Å).

## Conclusion

In this contribution, we have reported on the synthesis and characterization of the first representative of alkaline earth nitridoborophosphates, Ba_2_BP_7_N_14_. The synthesis was performed under HP/HT conditions (8 GPa, 1600 °C) using the multianvil technique, employing a combined nitride/azide route. The structure model refined from SCXRD data, was confirmed by PXRD, elemental analysis (EDX, CHNS), low‐cost crystallographic calculations (CHARDI, MAPLE) and further advanced microscopic and spectroscopic methods (STEM‐HAADF, EELS, ^31^P and ^11^B MAS NMR). Furthermore, the optical band gap (undoped sample), the luminescence behavior upon Eu^2+^ doping, and the thermal stability (25–950 °C) were investigated. Ba_2_BP_7_N_14_ crystallizes in the barylite‐1O polytype (MOD_1_, BaBe_2_Si_2_O_7_) and is isotypic to the nitridosilicatephosphates *AE*SiP_3_N_7_ (*AE*=Sr, Ba; *Pmn*2_1_). The crystal structure of the title compound exhibits a highly condensed mixed (B,P)−N 3D network of all‐side vertex‐sharing PN_4_ and mixed occupied (P_0.75_B_0.25_)N_4_ tetrahedra. The statistically disordered site was supported by EELS, STEM and NMR analyses as well as low‐cost crystallographic calculations. The B−P−N system has thus been successfully supplemented through the first quaternary alkaline earth metal representative. Moreover, the first isotypic representative of highly condensed, mixed tetrahedra‐based nitridophosphates is now accessible. This approach provides a foundation for examining the direct influence of B^+III^ vs. Si^+IV^ as *NFCs* on structure‐property relationships within a nitridophosphate‐based system, for the first time. Therefore, theoretical calculations based on density functional theory (DFT) can be used in future work.

## Experimental Section

**Synthesis of NH_4_N_3_**: According to Frierson *et al*. NH_4_N_3_ was synthesized by sublimation using stoichiometric amounts of NaN_3_ (Acros Organics, 99%) and dry NH_4_NO_3_ (Grüssing, 99%).^[50]^ The reactants were ground and the mixture was placed in a dry Schlenk tube. Only the lower part of the Schlenk tube was heated up to 200 °C for 12 h with open valve. During the reaction, the product was precipitated as colorless crystals at the cold end of the Schlenk tube. The phase purity of the product was confirmed by powder X‐ray diffraction and FTIR spectroscopy.

**Synthesis of Ba(N_3_)_2_**: The preparation of Ba(N_3_)_2_ was carried out according to a synthesis route for Ca(N_3_)_2_, which was modified and adapted to Ba(N_3_)_2_.^[51]^ A suspension of Ba(OH)_2_ ⋅ 8 H_2_O (Sigma Aldrich, ≥98%) and a threefold excess of NH_4_N_3_ was prepared in 200 mL water. The generated NH_3_ was boiled off, and BaCO_3_ impurities were filtered. After removing the solvent under reduced pressure, the product was obtained as a colorless crystalline solid. Phase purity was confirmed by powder X‐ray diffraction and FTIR spectroscopy.

**Synthesis of P_3_N_5_**: P_3_N_5_ was synthesized from P_4_S_10_ (ca. 8 g, Sigma Aldrich, 99.99%) with a constant flow of dried NH_3_ (Air Liquide, 5.0) according to Stock *et al*.^[52]^ Before starting the reaction, a silica tube and an inserted silica boat were dried in a tube furnace at 1000 °C *in vacuo*. After cooling the apparatus, P_4_S_10_ was placed in the silica boat and the silica tube was saturated with NH_3_ for 1 h. The apparatus was heated up to 850 °C (7 °C/min) and the temperature was kept for 4 h. The resulting orange product was washed with water, ethanol and acetone and was dried under reduced pressure (<10^−3^ mbar). The phase purity of the product was confirmed by powder X‐ray diffraction, FTIR spectroscopy and CHNS analysis.

**h‐BN**: h‐BN (abcr GmbH, 99%) was used without any further purification.

**High‐Pressure High‐Temperature Syntheses**: High‐pressure high‐temperature syntheses were performed in a 1000 t press (Voggenreiter, Mainleus, Germany) with a modified Walker‐type multianvil apparatus. The starting materials were ground in a glove box (MBraun, filled with argon, O_2_ <1 ppm, H_2_O<0.1 ppm) and the mixture was transferred to a crucible of h‐BN (HeBoSint® S100, Henze, Kempten, Germany). After sealing the crucible with an h‐BN cap, it was placed in two graphite furnaces (Schunk Kohlenstofftechnik GmbH, Gießen, Germany) and centered with two MgO spacers. This composition was placed in a ZrO_2_ sleeve (Cesima Ceramics, Wust‐Fischbeck, Germany), which was sealed on both sides with Mo plates. The ZrO_2_ sleeve acts as a thermal insulator and the Mo plates provide the electrical contact between the graphite furnaces and the apparatus. The filled and sealed ZrO_2_ sleeve was centered in the middle of a drilled MgO octahedron (doped with 5% Cr_2_O_3_, 18 mm edge length, Ceramic Substrates & Components Ltd, Isle of Wight, UK), which served as pressure medium. The prepared octahedron was placed in an assembly of eight Co‐doped WC cubes (7% Co, truncated edge length 11 mm, Hawedia, Marklkofen, Germany) with truncated edges. Additional attached pyrophyllite gaskets (Ceramic Substrates & Components, Isle of Wight, UK) were used to separate the eight WC cubes and prevent outflow of the octahedron during the synthesis. More detailed information on the high‐pressure setup can be found in literature.[^53^, ^54^]

**Synthesis of Ba_2_BP_7_N_14_**: Ba_2_BP_7_N_14_ was prepared from stoichiometric amounts of Ba(N_3_)_2_, P_3_N_5_ and h‐BN by a high‐pressure high‐temperature synthesis as described above. The mixture of the starting materials was compressed to 8 GPa and then heated to 1600 °C within 35 min. These conditions were kept for 2 h. After cooling to room temperature within 4 h, the system was decompressed. The colorless product was obtained after removing the crucible.

**Synthesis of BaP_2_N_4_**: BaP_2_N_4_ was synthesized by a high‐pressure high‐temperature synthesis as described above, based on a procedure by Karau *et al*.^[55]^ The reactant mixture of stoichiometric amounts of Ba(N_2_)_3_ and P_3_N_5_ was compressed to 8 GPa. After heating the reaction up to 1400 °C within 35 min, the pressure and temperature were kept for 1 h. Within 2 h, the synthesis was cooled to room temperature and decompressed to ambient pressure. After removing the crucible, the colorless crystalline solid was obtained. Phase purity of the prepared solid was confirmed by powder X‐ray diffraction.

**Single‐Crystal X‐ray Diffraction (SCXRD)**: Single‐crystal X‐ray diffraction data of Ba_2_BP_7_N_14_ were collected on a Bruker D8 Venture TXS diffractometer (Mo‐K*α* radiation *λ*=0.71073 Å, rotating anode, multilayer monochromator). Indexing, integration and multi‐scan absorption correction were performed using the APEX3 software package.[^56^, ^57^, ^58^] The structure was solved by direct methods using SHELXT and refined by full‐matrix least‐square method using SHELXL.[^59^, ^60^] All atoms (Ba, B, P, N) were refined anisotropically. The resulting space group was checked with PLATON.[^61^, ^62^] The results were visualized using VESTA software.^[63]^

**Powder X‐ray Diffraction (PXRD)**: A STOE Stadi P diffractometer (STOE & Cie GmbH, Darmstadt, Germany) with Mo‐K*α*_1_ radiation, Ge(111) monochromator, and a MYTHEN 1K Si strip detector in modified parafocusing Debye‐Scherrer geometry, was used to record powder X‐ray diffraction patterns. The ground sample was filled and sealed in a glass capillary (*d*=0.3 mm, Hilgenberg GmbH, Malsfeld, Germany). Rietveld refinements were performed using the TOPAS software.[^64^, ^65^] Temperature‐dependent powder X‐ray diffraction was carried out on a STOE Stadi P diffractometer (STOE & Cie GmbH, Darmstadt, Germany, Mo‐K*α*_1_ radiation, Ge(111) monochromator, STOE resistance graphite furnace and IP‐PSD detector). The sample was ground and filled in air in a quartz glass capillary (*d*=0.3 mm, Hilgenberg GmbH, Malsfeld, Germany). Measurements were performed between 25–950 °C with 25 °C increments.

**Scanning Electron Microscopy (SEM) and Energy‐Dispersive X‐ray Spectroscopy (EDX)**: SEM imaging and EDX analysis were performed on a Dualbeam Helios Nanolab G3 UC (FEI, Hillsboro, OR, USA) equipped with an X‐Max 80 SDD detector (Oxford Instruments, Abingdon, UK). The sample was prepared on an adhesive carbon pad at ambient conditions and coated with carbon using an electron beam evaporator (BAL‐TEC MED 020, Bal Tec AG). EDX data were analyzed using the Aztec software.^[66]^

**CHNS Analysis**: A Vario Micro Cube device (Elementar, Langenselbold, Germany) was used for quantitative elemental analysis.

**Scanning Transmission Electron Microscopy (STEM) and Electron Energy Loss Spectroscopy (EELS)**: STEM and EELS analyses were performed on a Titan Themis 300 (FEI, USA) electron microscope equipped with a X‐FEG electron source, a US1000XP/FT camera system (Gatan, Germany), a post‐column filter (Enfinium ER‐799, Gatan, USA), and a windowless 4‐quadrant Super‐X EDX detector. The device was operated at 300 kV acceleration voltage. Bright field images and selected area electron diffraction diagrams were recorded with a 4k×4k Ceta CMOS camera (FEI, USA). For STEM‐HAADF (high angle annular dark field) imaging a HAADF detector (inner half angle 33 mrad for 245 mm camera length, semiconvergence angle 16.6 mrad, 50 μm aperture, FEI, USA) was used. EELS was performed with a Gatan Enfinium spectrometer (convergence angle of 16.6 mrad and a collection angle of 44 mrad). For preparation, the sample was ground in absolute ethanol. A drop of the received suspension was applied on a TEM Cu grid coated with carbon‐film (Plano GmbH, Wetzlar, Germany). The prepared grid was placed on a double‐tilt holder. The following softwares were used for STEM data evaluation: Digital Micrograph, ProcessDiffraction 7, JEMS and Velox v3.0.[^67^, ^68^, ^69^, ^70^]

**Solid‐State NMR Spectroscopy**: ^31^P, ^31^P{^1^H} cross‐polarization and ^11^B spin‐echo MAS NMR experiments were performed on an Avance III 500 spectrometer (Bruker, Karlsruhe, Germany) and indirectly referenced to ^1^H in 100% tetramethylsilane (TMS) at −0.1240 ppm. For the measurements, the sample was ground and loaded in a 2.5 mm ZrO_2_ rotor and NMR spectra were collected at 20 kHz spinning frequency. Deconvolution of the ^11^B spin‐echo MAS NMR spectrum was performed using the Igor Pro software.^[71]^

**UV/Vis Spectroscopy**: Diffuse reflectance spectrum of an undoped sample was recorded in the range of 240–800 nm on a UV‐Vis spectrophotometer (V‐650, JASCO, Gross‐Umstadt, Germany). The device was equipped with a deuterium (190–350 nm) and a halogen (330–900 nm) lamp, a single monochromator (1200 lines/mm), and a photomultiplier tube detector.

**Luminescence Spectroscopy**: Luminescence measurements of bulk material (doped with Eu^2+^) were carried out at room temperature on a spectrofluorimeter equipped with a 75 W Xe‐lamp model A‐1010B, a monochromator model 101 and a fiber spectrometer AvaSpec‐2048 TEC‐USB1.0 (Avantes). For the measurements, the powder was placed on a PTFE sample holder which was positioned inside an in‐house built system, based on a 5.3” integration sphere. Background corrections were performed using a BaSO_4_ white standard.

## Conflict of Interests

The authors declare no conflict of interest.

## Supporting information

As a service to our authors and readers, this journal provides supporting information supplied by the authors. Such materials are peer reviewed and may be re‐organized for online delivery, but are not copy‐edited or typeset. Technical support issues arising from supporting information (other than missing files) should be addressed to the authors.

Supporting Information
