# Supplementary material for: Ba2BP7N14 – A Quaternary Alkaline Earth Nitridoborophosphate with a Mixed 3D Network Structure
Source: Chemistry. 2025 Feb 21;31(20):e202404755. doi: 10.1002/chem.202404755 (PMC11973858; doi:10.1002/chem.202404755)
Supplement: Supplementary file 1 — Supporting Information [file CHEM-31-e202404755-s001.pdf]

Chemistry–A European Journal

Supporting Information

$Ba_2BP_7N_{14}$ – A Quaternary Alkaline Earth Nitridoborophosphate with a Mixed 3D Network Structure

Amalina T. Buda, Reinhard M. Pritzl, Monika M. Pointner, Jennifer Steinadler, and Wolfgang Schnick*

Table of Contents

Previous Synthesis Experiments 2
Synthesis 3
Energy-Dispersive X-ray (EDX) Measurements 4
CHNS Analysis 4
Crystallographic Data 5
Rietveld Refinement 6
Charge Distribution (CHARDI) Calculations 7
Madelung Part of the Lattice Energy (MAPLE) Calculations 7
Electron Energy Loss Spectroscopy (EELS) 8
Scanning Transmission Electron Microscopy (STEM) 9
Solid-State Magic-Angle Spinning (MAS) NMR Spectroscopy 11
Temperature-Dependent Powder X-ray Diffraction (PXRD) 13
UV/Vis Spectroscopy 14

Previous Synthesis Experiments

In order to investigate the influence and necessity of h-BN on the formation of the title compound identified by SCXRD, previous synthesis experiments were performed at 6 GPa and 1600 °C using a Walker-type multianvil press. During the experiments, a Pt-inlay was used to completely separate the reactant mixture from the h-BN crucible, which is state of the art for such high-pressure syntheses.[53] This procedure allows for determining only the influence of the added h-BN. For the initial synthesis, no h-BN powder was added to the mixture of $Ba(N_3)_2$ and P_3N_5. Based on the resulting Rietveld refinement, no evidence of the formation of the target compound can be observed (Figure S1). In contrast, the compounds BaP_2N_4, $Ba_3(PO_4)_2$ and PtP_2 were identified. In the subsequent synthesis, the addition of h-BN powder to the reaction mixture resulted in the formation of the target compound $Ba_2BP_7N_{14}$, as well as BaP_2N_4 and $Ba_3(PO_4)_2$ (Figure S2). Since the target compound was obtained exclusively through the utilization of h-BN powder, it can be concluded that h-BN is essential for its formation.

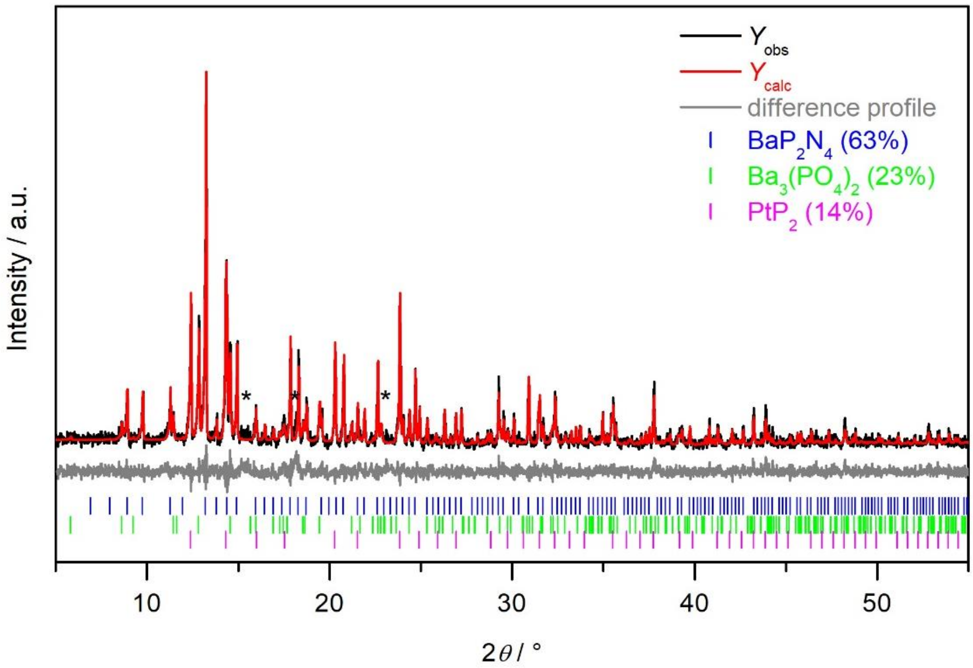

Figure S1. Rietveld refinement of the synthesized product using a Pt-inlay, without adding h-BN powder to the reaction mixture of $Ba(N_3)_2$ and P_3N_5. Observed (black line) and calculated (red line) powder X-ray diffraction patterns, difference profile (gray line), positions of Bragg reflections of BaP_2N_4 (vertical blue bars), $Ba_3(PO_4)_2$ (vertical green bars) and PtP_2 (vertical magenta bars); R_p = 0.124, R_{wp} = 0.162, X^2 = 0.878. Reflections marked with an asterisk originate from an unknown side phase.

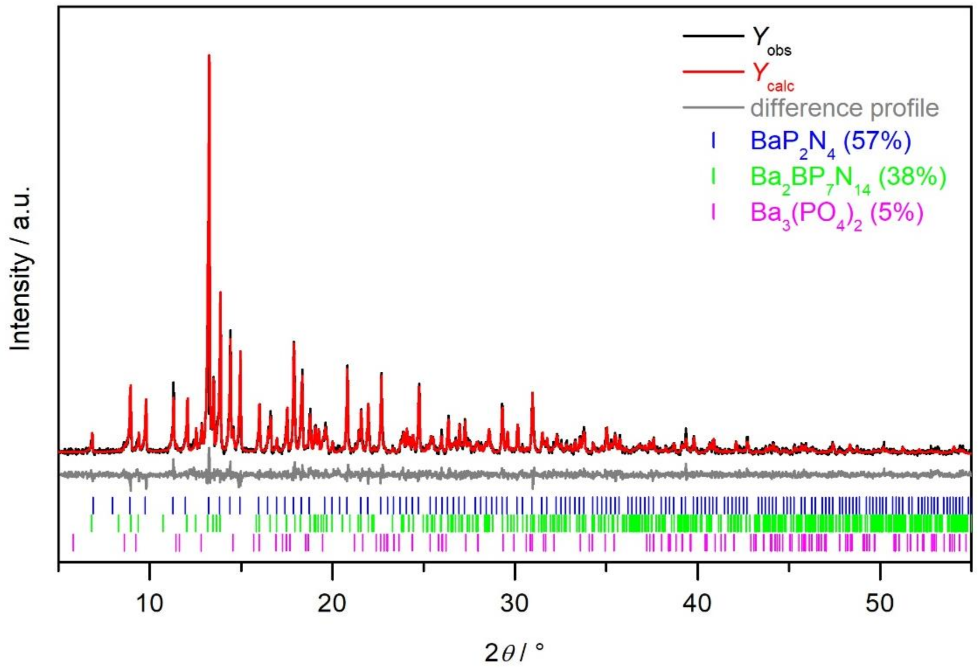

Figure S2. Rietveld refinement of the synthesized product using a Pt-inlay, with the addition of h-BN powder to the reaction mixture of $Ba(N_3)_2$ and P_3N_5. Observed (black line) and calculated (red line) powder X-ray diffraction patterns, difference profile (gray line), positions of Bragg reflections of BaP_2N_4 (vertical blue bars), the target compound $Ba_2BP_7N_{14}$ (vertical green bars, R_{Bragg} = 0.0302) and $Ba_3(PO_4)_2$ (vertical magenta bars); R_p = 0.108, R_{wp} = 0.144, X^2 = 0.932.

Synthesis

Table S1. Weighed portions of the starting materials for the optimized synthesis of $Ba_2BP_7N_{14}$. $EuCl_2$ was used as a dopant for $Ba_2BP_7N_{14}$:Eu^{2+}.

Compound	Starting material	Amount / mg	Amount of substance / mmol
	$Ba(N_3)_2$	31.7	0.143
$Ba_2BP_7N_{14}$	P_3N_5	27.2	0.167
	h-BN	1.8	0.073

Energy-Dispersive X-ray (EDX) Measurements

Table S2. EDX measurements of $Ba_2BP_7N_{14}$ are given in atomic percent. Standard deviations are given in parentheses. No other atoms than Ba, B, P, N and O were detected.

#	**Ba**	**B**	**P**	**N**	**O**
1	8.2	8.1	29.7	48.7	5.3
2	7.6	9.7	27.6	50.0	5.0
3	5.2	9.4	20.4	57.6	7.4
4	7.4	6.3	27.2	56.1	2.9
5	11.0	6.7	32.7	45.9	3.7
6	9.1	6.0	30.0	49.4	5.5
7	6.7	10.2	24.4	53.7	5.0
8	8.9	6.1	31.0	51.1	2.8
**calc.**	8.3	4.2	29.2	58.3	/
**average**	8(2)	8(3)	28(5)	52(4)	5(2)
**normalized (Ba)**	2	2	7	13	1

CHNS Analysis

Table S3. Results of the CHNS analysis and theoretical values for the possible sum formulas "$Ba_2P_7N_{11}O_3$", "$Ba_2P_7N_{11}(NH)_3$" and $Ba_2BP_7N_{14}$. The elements H, C and S were not detected (wt% = 0).

	**H / wt%**	**N / wt%**
**exp.**	**0**	**27.4**
**"$Ba_2P_7N_{11}O_3$"(theo.)**	0	22.2
**"$Ba_2P_7N_{11}(NH)_3$"(theo.)**	0.44	28.4
**$Ba_2BP_7N_{14}$(theo.)**	0	28.1

Crystallographic Data

Table S4. Wyckoff positions, coordinates, equivalent thermal displacement parameters, and site occupancies of $Ba_2BP_7N_{14}$ from refinement of single-crystal XRD data. Standard deviations are given in parentheses.

Atom	Wyckoff	*x*	*y*	*z*	U_{eq}	*s.o.f.*
Ba1	2*a*	0	0.29013(5)	0.00000(2)	0.01516(6)	1
P1	4*b*	0.37955(5)	0.18022(12)	0.0180(3)	0.00721(13)	1
P2|B2	4*b*	0.25081(6)	0.32066(14)	0.4998(4)	0.00766(11)	0.75|0.25
N1	4*b*	0.1395(2)	0.1325(5)	0.4485(6)	0.0143(6)	1
N2	2*a*	0	0.7264(9)	0.4079(9)	0.0156(7)	1
N3	4*b*	0.3677(2)	0.2347(6)	0.3478(6)	0.0122(4)	1
N4	4*b*	0.2184(2)	0.6318(6)	0.3550(6)	0.0122(4)	1

Table S5. Anisotropic displacement parameters (U_{ij} / Å^2) of $Ba_2BP_7N_{14}$ from refinement of single-crystal XRD data. Standard deviations are given in parentheses.

Atom	U_{11}	U_{22}	U_{33}	U_{12}	U_{13}	U_{23}
Ba1	0.02052(10)	0.01429(9)	0.01066(9)	0	0	0.00087(18)
P1	0.0082(2)	0.0059(2)	0.0076(4)	0.00088(17)	−0.0003(3)	−0.0003(3)
P2|B2	0.0076(3)	0.0082(3)	0.0073(3)	−0.0010(2)	−0.0002(6)	−0.0003(7)
N1	0.0153(10)	0.0096(9)	0.0180(17)	0.0044(8)	−0.0007(9)	0.0057(8)
N2	0.0067(13)	0.0230(19)	0.0170(15)	0	0	−0.0072(14)
N3	0.0128(10)	0.0129(10)	0.011(1)	−0.0014(9)	−0.0017(8)	−0.0017(8)
N4	0.0083(9)	0.0116(10)	0.0168(11)	0.0040(8)	−0.0010(8)	−0.0019(9)

Table S6. Interatomic distances of $Ba_2BP_7N_{14}$ from single-crystal refinement. Standard deviations are given in parentheses.

Atom1–Atom2		distance / Å	Atom1–Atom2	distance / Å
Ba1—N1	2x	2.815(3)	P1—N3	1.593(3)
Ba1—N1	2x	3.200(3)	P1—N4	1.684(3)
Ba1—N2		2.896(4)	P2|B2—N1	1.644(3)
Ba1—N3	2x	2.924(3)	P2|B2—N3	1.632(3)
Ba1—N3	2x	3.124(3)	P2|B2—N4	1.729(3)
P1—N1		1.597(3)	P2|B2—N4	1.739(4)
P1—N2		1.603(2)		

Table S7. Interatomic angles of $Ba_2BP_7N_{14}$ from refinement of the single-crystal data. Standard deviations are given in parentheses.

**Atom1–Atom2–Atom3**	**angle / °**	**Atom1–Atom2–Atom3**	**angle / °**
N3—P1—N1	110.69(15)	N3—P2|B2—N1	118.93(16)
N3—P1—N2	110.5(2)	N3—P2|B2—N4	104.44(16)
N1—P1—N2	109.9(2)	N1—P2|B2—N4	105.21(14)
N3—P1—N4	107.17(15)	N3—P2|B2—N4	106.27(14)
N1—P1—N4	109.85(15)	N1—P2|B2—N4	113.07(17)
N2—P1—N4	108.61(18)	N4—P2|B2—N4	108.18(11)

Rietveld Refinement Data

Table S8. Crystallographic data from Rietveld refinement of $Ba_2BP_7N_{14}$. Standard deviations are given in parentheses.

**Formula**	**$Ba_2BP_7N_{14}$**
Crystal system	orthorhombic
Space group	*Pmn*2$_1$ (no. 31)
Lattice parameters / Å	a = 11.93489(14)<br>b = 4.92173(7)<br>c = 4.67542(7)
Cell volume / Å^3	274.635(6)
Radiation λ / Å	0.7093 (Mo-Kα_1)
2θ-range / °	5 < 2θ < 60
Data points	3668
Number of parameters (thereof background)	44(14)
Background function	Shifted Chebyshev
R values	R_{Bragg} = 0.0173<br>R_p = 0.0452<br>R_{wp} = 0.0594
Goodness of fit	1.414

Table S9. Wyckoff positions, coordinates, isotropic thermal displacement parameters, and site occupancies of $Ba_2BP_7N_{14}$ from Rietveld refinement. Standard deviations are given in parentheses.

Atom	Wyckoff	*x*	*y*	*z*	U_{eq}	*s.o.f.*
Ba1	2*a*	0	0.29040(16)	0.0005(4)	1.53(3)	1
P1	4*b*	0.37899(15)	0.1741(5)	0.0247(9)	0.49(5)	1
P2	4*b*	0.2511(2)	0.3239(5)	0.494(2)	0.49(5)	0.75
B2	4*b*	0.2511(2)	0.3239(5)	0.494(2)	0.49(5)	0.25
N1	4*b*	0.1396(5)	0.1353(11)	0.4452(17)	0.49(5)	1
N2	2*a*	0	0.733(2)	0.4060(16)	0.49(5)	1
N3	4*b*	0.3681(5)	0.2395(15)	0.3546(10)	0.49(5)	1
N4	4*b*	0.2191(6)	0.6348(13)	0.3530(13)	0.49(5)	1

Charge Distribution (CHARDI) Calculations

Table S10. Results of CHARDI calculations (mean total charges and coordination numbers (*CN*)) and theoretical values of the atom sites in the crystal structure of $Ba_2BP_7N_{14}$.

Atom site	$charge_{CHARDI}$ / $charge_{theo.}$	CN_{CHARDI} / $CN_{theo.}$
Ba1	+1.94 / +2.00	8.01 / 9
P1	+5.00 / +5.00	3.92 / 4
P2|B2	+4.53 / +4.50	3.87 / 4
N1	−3.09 / −3.00	
N2	−2.92 / −3.00	
N3	−3.14 / −3.00	
N4	−2.80 / −3.00	

Madelung Part of the Lattice Energy (MAPLE) Calculations

Table S11. Results of MAPLE calculations for $Ba_2BP_7N_{14}$. Total MAPLE values are given of the formal reaction of BaP_2N_4 and BP_3N_6 forming $Ba_2BP_7N_{14}$.

**2 BaP_2N_4 + BP_3N_6**		→	**$Ba_2BP_7N_{14}$**
2x BaP_2N_4	111938 kJ·mol^{-1}		199896 kJ·mol^{-1}
BP_3N_6[26]	90556 kJ·mol^{-1}		
∑	202494 kJ·mol^{-1}		**1.3% difference**

Electron Energy Loss Spectroscopy (EELS)

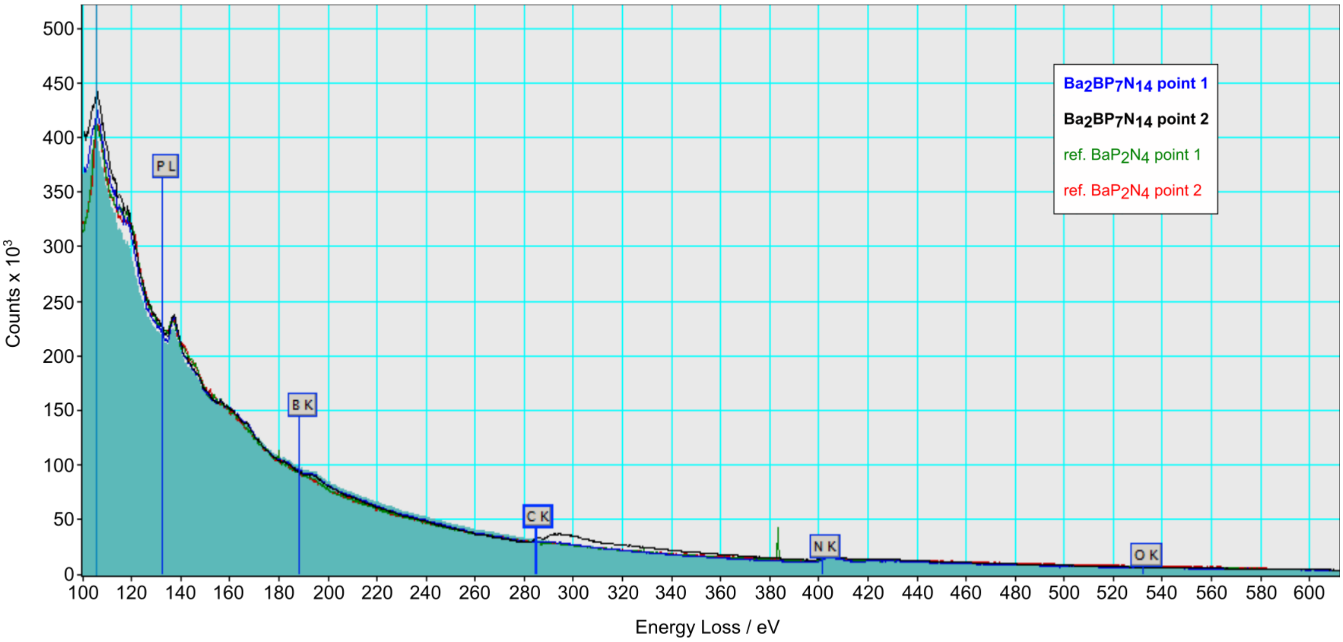

Figure S3. EELS spectra in the range of 100–610 eV, messured at two points of $Ba_2BP_7N_{14}$ crystallites (blue and black line) and at two points of BaP_2N_4 crystallites (reference component, green and red line). The positions of the theoretical edges are marked by vertical lines. The $Ba_2BP_7N_{14}$ and BaP_2N_4 spectra each show a P-$L_{2,3}$ and N-K edge at ~137 eV and ~406 eV energy loss, respectively. In the spectra of $Ba_2BP_7N_{14}$, an additional energy loss of ~194 eV can be observed at the B-K edge. Detections of carbon, can be attributed to the sample support. Due to the insufficient resolution of the measured spectra at the O edge (theo. 532 eV), it was not possible to ascertain whether the target compound was contaminated by oxygen.

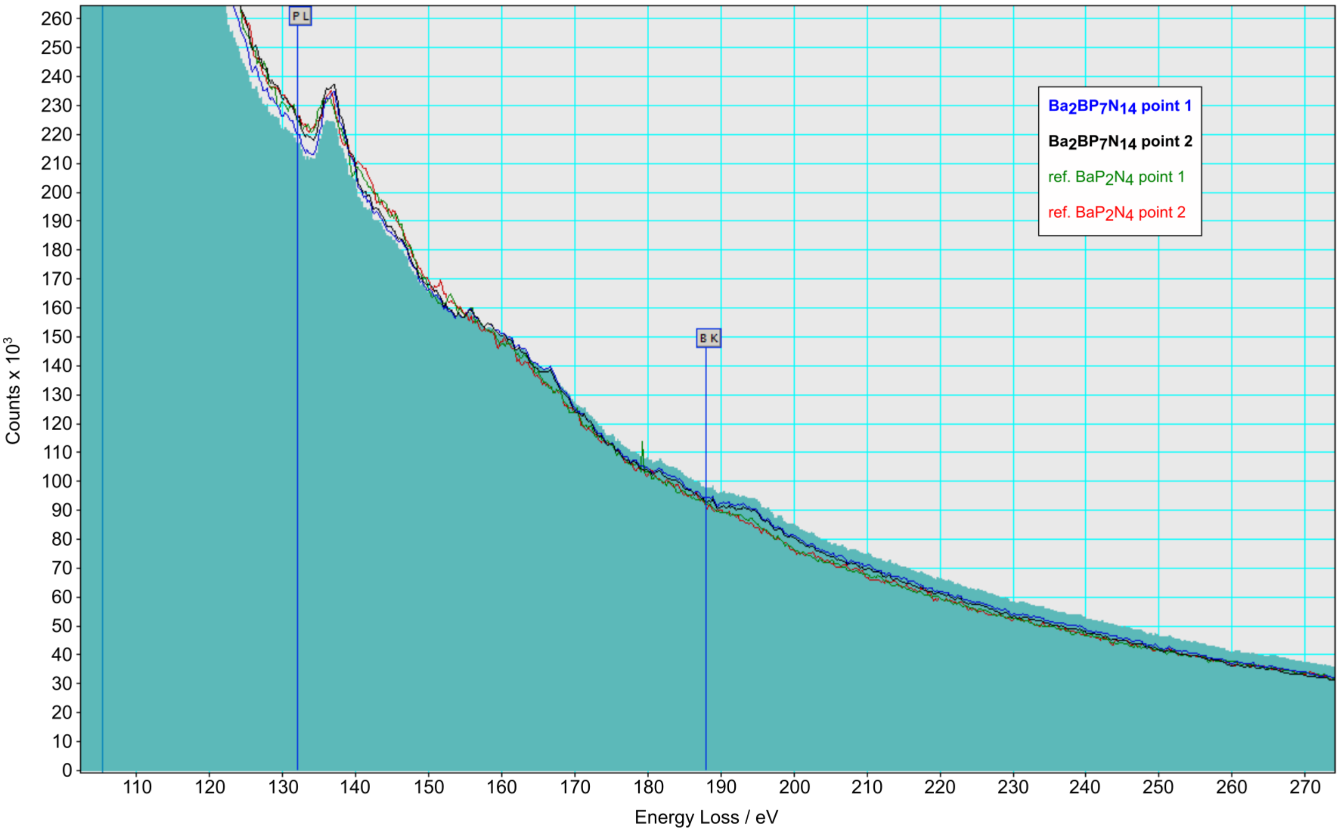

Figure S4. Extended EELS spectra in the range of 100–275 eV, measured at two points of $Ba_2BP_7N_{14}$ crystallites (blue and black line) and at two points of BaP_2N_4 crystallites (reference component, green and red line). The positions of the theoretical edges are marked by a vertical line. The $Ba_2BP_7N_{14}$ and BaP_2N_4 spectra each show a P-$L_{2,3}$ at ~137 eV energy loss. In the spectra of $Ba_2BP_7N_{14}$, an additional energy loss of ~194 eV can be observed at the B-K edge.

Scanning Transmission Electron Microscopy (STEM)

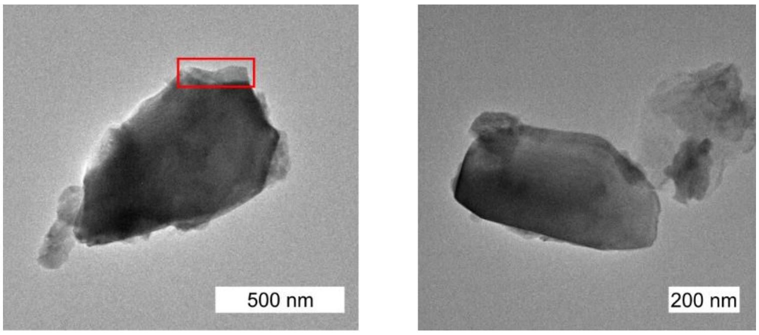

Figure S5. Bright-field images of $Ba_2BP_7N_{14}$ crystallites. The range for STEM analysis is highlighted with a red rectangle (left).

Table S12. Normalized intensity maxima of the positions P1 and P2/B2 of $Ba_2BP_7N_{14}$ examined by STEM-HAADF analysis. The intensity histograms of the atomic positions Ba1–P1–P2/B2–P2/B2–P1–Ba1 were examined in different areas of the zone axis [001]. The obtained intensity maxima were normalized to Ba intensities.

	**P1**	**P2|B2**	**P2|B2**	**P1**
**1**	0.23	0.16	0.11	0.20
**2**	0.21	0.17	0.09	0.20
**3**	0.23	0.16	0.14	0.20
**4**	0.21	0.15	0.14	0.17
**5**	0.24	0.15	0.13	0.19
**6**	0.24	0.18	0.19	0.20
**7**	0.22	0.16	0.13	0.21
**8**	0.21	0.16	0.16	0.16

Table S13. Average values and percentage ratio of the normalized intensity maxima of the positions P1 and P2/B2 of $Ba_2BP_7N_{14}$ examined by STEM-HAADF analysis.

	**P1**	**P2|B2**
**Average**	0.21(2)	0.15(2)
**Percentage ratio**	100%	71%

Determination of the theoretical intensity maxima of the fully occupied P1 site of $Ba_2BP_7N_{14}$ with respect to the Z contrast of Z^2 ($Z_P = 15$).[42]

$$I_{theo}(P1) = 1 \cdot Z(P)^2 = 1 \cdot 15^2 = 225\ (\equiv 100\%) \qquad (S1)$$

Determination of the experimental intensity maxima of the atomic position P2/B2 $Ba_2BP_7N_{14}$ with respect to the determined percentage ratio of 71% in STEM-HAADF analysis.

$$I_{exp}(P2/B2) = (I_{theo}(P1) \cdot 71\%)/100\% = (225 \cdot 71\%)/100\% \approx 160 \qquad (S2)$$

Determination of the occupation ratio of the mixed P2/B2 side observed by STEM-HAADF analysis with respect to the Z contrast of Z^2 ($Z_P = 15$, $Z_B = 5$).[42]

$$I_{exp}(P2/B2) = x \cdot Z(P)^2 + (1–x) \cdot Z(B)^2 \qquad (S3)$$

$$160 = x \cdot 15^2 + (1–x) \cdot 5^2$$

$$\mathbf{0.7 \approx x \quad \rightarrow P_{0.7}B_{0.3}}$$

Solid-State Magic-Angle Spinning (MAS) NMR Spectroscopy

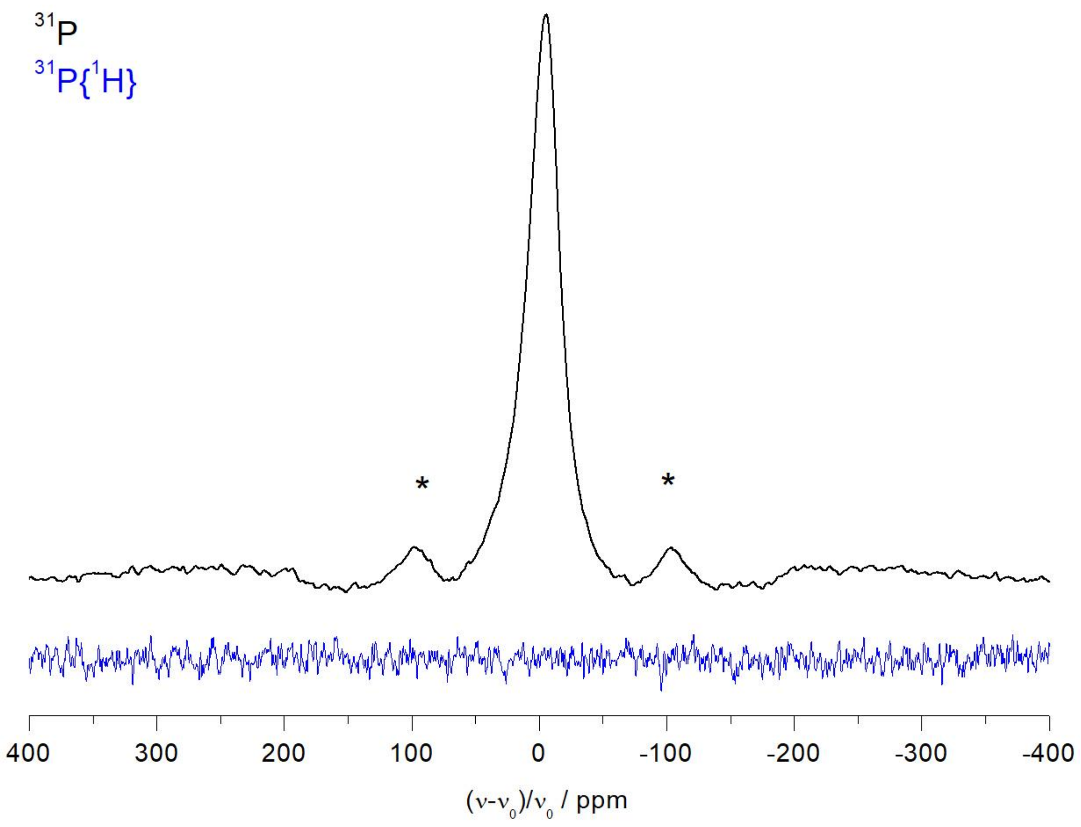

Figure S6. ^{31}P MAS NMR (black line) and $^{31}P\{^{1}H\}$ cross polarization MAS NMR (blue line) spectra of $Ba_2BP_7N_{14}$. Rotational sidebands are marked with asterisks (*).

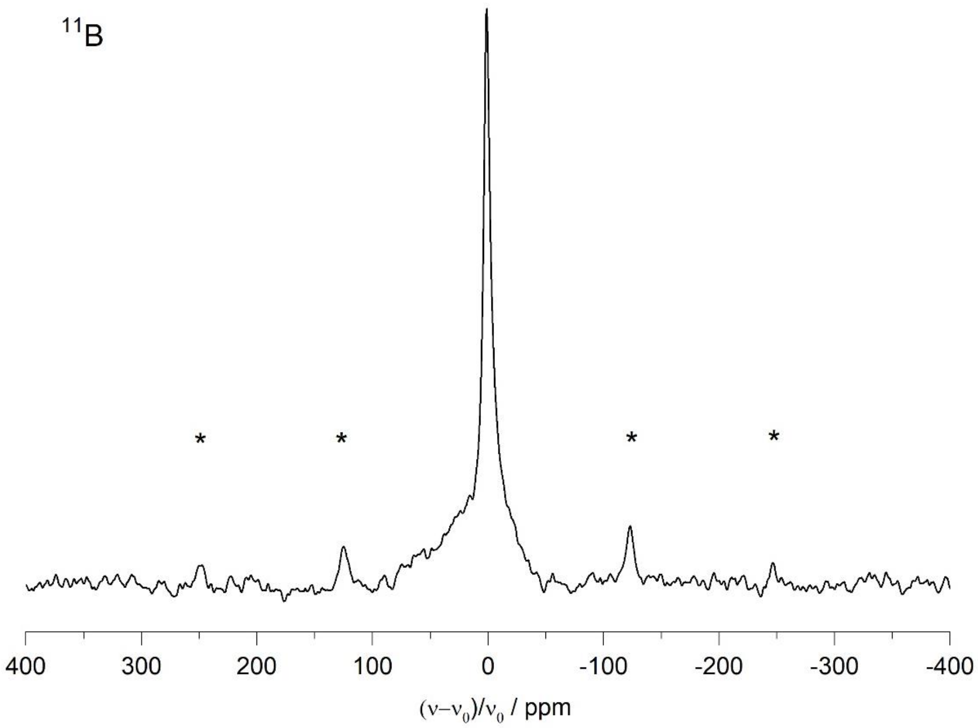

Figure S7. ^{11}B spin-echo MAS NMR spectrum of $Ba_2BP_7N_{14}$. Rotational sidebands are marked with asterisks (*).

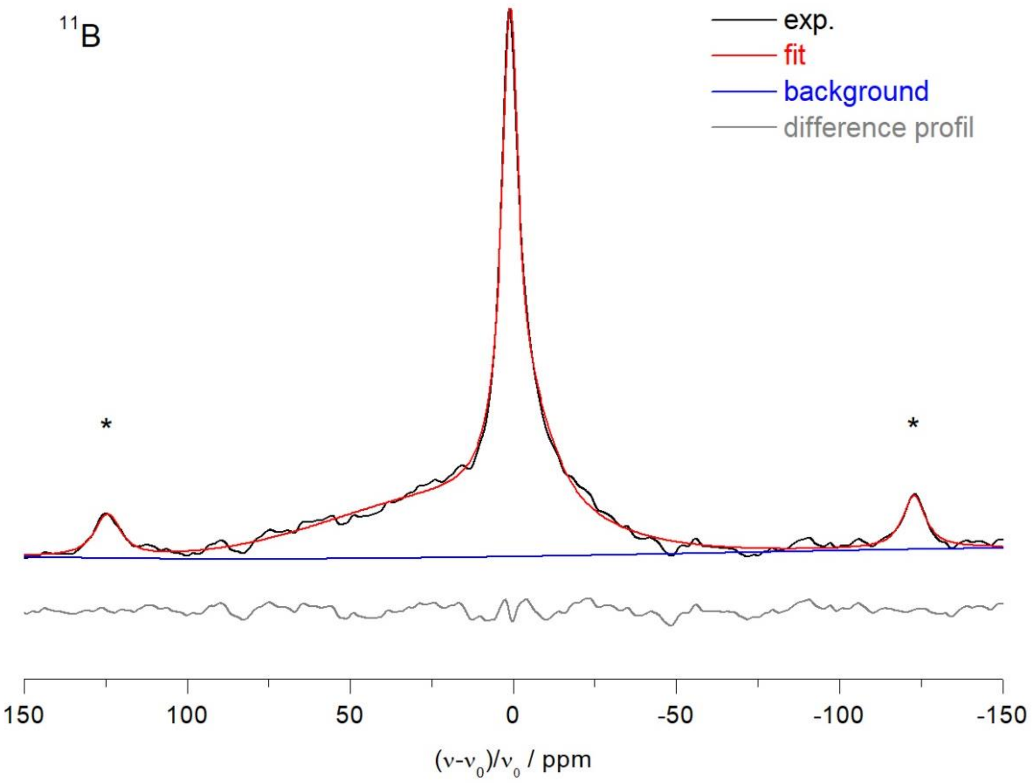

Figure S8. Deconvolution fit of the ^{11}B signals from ^{11}B spin-echo MAS NMR spectrum of $Ba_2BP_7N_{14}$. The figure illustrates the measured (black line) and the fitted (envelope of Voigt functions, red line) NMR spectrum, the fitted background (blue line) and the difference profile (gray line). Rotational sidebands are marked with asterisks (*).

Table S14. Comparison of the area ratio of the ^{11}B signals (obtained by deconvolution of the ^{11}B spin-echo MAS NMR spectrum) and the molar ratio of the B atoms (obtained by Rietveld refinement of the PXRD data; 6 wt% c-BN and 94 wt% $Ba_2BP_7N_{14}$) of c-BN and $Ba_2BP_7N_{14}$.

	**Integral (^{11}B signal)**	**Amount of B (Rietveld refinement) / mmol**
**c-BN**	5.09(8) · 10^7	0.24
**$Ba_2BP_7N_{14}$**	3.0(2) · 10^7	0.13
**Ratio (c-BN/ $Ba_2BP_7N_{14}$)**	**1.7**	**1.8**

Temperature-Dependent Powder X-ray Diffraction

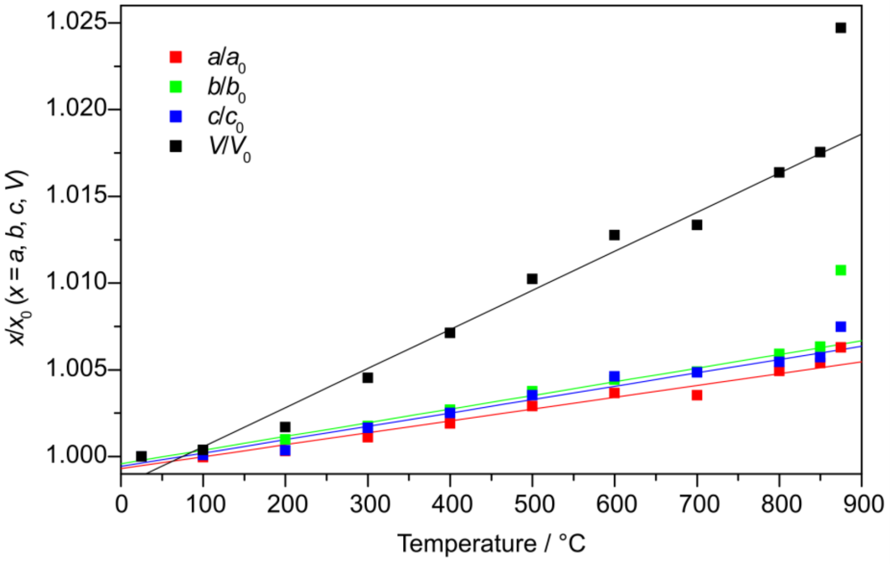

Figure S9. Temperature-dependent behavior of the lattice parameters (*a*, *b*, *c*) and the cell volume (*V*) of $Ba_2BP_7N_{14}$, determined from PXRD pattern measured in air. Linear dependencies were observed in the range of 25-850 °C. The following expansion coefficients were optained: α_a = 6.9(4), α_b = 7.9(2), α_c = 7.7(4), α_V = 22.5(9) ppm·K^{-1}. Non-linear behavior of the parameters occur at 875 °C, indicating the beginning of the decomposition process of $Ba_2BP_7N_{14}$.

UV/Vis Spectroscopy

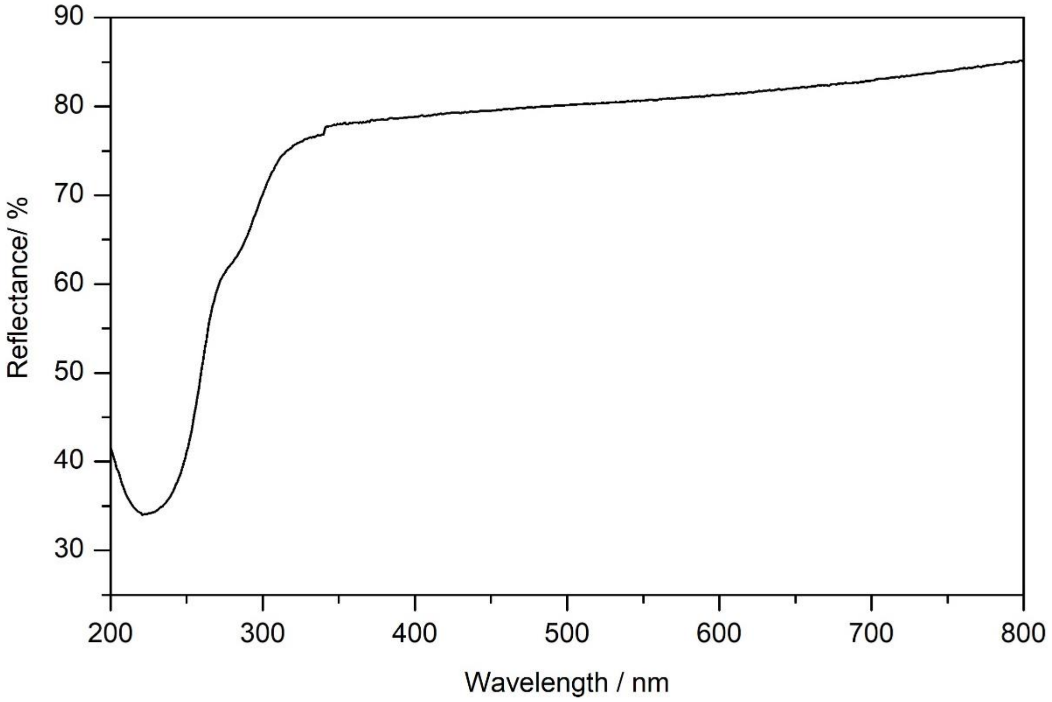

Figure S10. Diffuse reflectance spectrum of $Ba_2BP_7N_{14}$.
